# Cognitive dysfunction of patients infected with SARS-CoV-2 omicron variant in Shanghai, China

**DOI:** 10.1186/s40035-023-00357-x

**Published:** 2023-05-24

**Authors:** Ping Yuan, Yong Bi, Yu Luo, Quan Tao, Sugang Gong, Yi Wang, Lize Xiong, Xiaohuan Xia, Jialin C. Zheng

**Affiliations:** 1 Translational Research Institute of Brain and Brain-Like Intelligence, Shanghai Fourth People’s Hospital Affiliated to Tongji University School of Medicine, Shanghai, 200081 China; 2 Department of Cardio-Pulmonary Circulation, Shanghai Pulmonary Hospital, School of Medicine, Tongji University, Shanghai, 200081 China; 3 Center for Translational Neurodegeneration and Regenerative Therapy, Tongji Hospital Affiliated to Tongji University, Shanghai, 200081 China; 4 Department of Anesthesiology and Perioperative Medicine, School of Medicine, Shanghai Fourth People’s Hospital Affiliated to Tongji University, Shanghai, 200081 China; 5 Department of Radiology, Shanghai Fourth People’s Hospital Affiliated to Tongji University School of Medicine, Shanghai, 200081 China; 6 Shanghai Frontiers Science Center of Nanocatalytic Medicine, Tongji University School of Medicine, Shanghai, 200081 China; 7 Center for Translational Neurodegeneration and Regenerative Therapy, Yangzhi Rehabilitation Hospital Affiliated to Tongji University, Shanghai, 200081 China; 8 Collaborative Innovation Center for Brain Science, Tongji University, Shanghai, 200081 China; 9 Clinical Research Center for Anesthesiology and Perioperative Medicine, Tongji University, Shanghai, 200081 China

Previous studies have reported that nearly one-fourth of coronavirus disease 2019 (COVID-19) survivors suffer from persistent cognitive impairment, including impaired memory, attention, concentration, executive function, and speed of information processing [1–3]. Omicron is the latest severe acute respiratory syndrome coronavirus 2 (SARS-CoV-2) variant of concern (VOC) reported in November 2021 [4], characterized by a large number of spike mutations, great transmissibility in the presence of other VOCs, and an ability to spread in populations with high level of immunity [4]. However, whether patients infected with Omicron COVID-19 variant develop cognitive impairment, similar to those infected with other VOCs, remains unclear.

In this cross-sectional cohort study, we enrolled 215 patients (142 asymptomatic and 73 mild without pneumonia) infected with SARS-CoV-2 Omicron BA.2.2.1 variant in Shanghai Fourth People’s Hospital from April 2022 to August 2022. All patients were diagnosed as infected by Omicron BA.2.2.1 variant according to the Diagnosis and Treatment Protocol for Novel Coronavirus Pneumonia (Trial Version 9). Patients with previous or present cerebral hemorrhage, traumatic brain injury or other neurological or psychiatric diseases, speech impairment, severe visual and hearing impairment, malignant tumor, alcoholism, drug abuse, and psychotropic substance abuse were excluded. Age- and gender-matched healthy control individuals (*n* = 215) were enrolled from the general Medical Examination Center in Shanghai Pulmonary Hospital between April 2022 and August 2022. This study was approved by ethical committees of both hospitals, and written informed consent has been given by all the participants.

Cognitive function was measured with Mini-Mental State Examination (MMSE) and Montreal Cognitive Assessment (MoCA) by a trained, professional person who implements the questionnaire regularly. MoCA has more testing of executive function and abstract ability and puts more weight on memory, attention-computation, and language function, while MMSE puts more weight to time and place orientation tests. The combination of MMSE and MoCA tests can grasp a certain aspect of cognitive dysfunction in most of the patients. The two questionnaires were completed within 6 h after the patient was confirmed to be positive for COVID-19 infection. The control subjects completed the two questionnaires within 6 h after they were confirmed to be negative for COVID-19 infection. Data were analyzed with the Mann–Whitney U test using SPSS 26.0. *P* < 0.05 was considered statistically significant.

Patients infected with Omicron variant showed comparable total scores of MMSE and MoCA to control subjects (Fig. 1a). Importantly, the COVID-19 patients over 50 years (61.9 ± 7.4 years, *n* = 100), but not those younger than 50 years (34.2 ± 9.5 years, *n* = 115), showed significantly lower total scores of MoCA and MMSE than controls (62.0 ± 7.3 years, *n* = 100; 34.3 ± 8.8 years, *n* = 115) (both *P* < 0.05, Fig. 1b). In addition, the female patients over 50 years (*n* = 60 in both groups) had significantly lower total scores of MMSE and MoCA than controls (both *P* < 0.05, Fig. 1c), while male patients aged over 50 (*n* = 40 in both groups) did not show significantly lower total scores of MMSE and MoCA compared with controls (Fig. 1c). Specifically, patients over 50 years infected with the Omicron variant demonstrated lower scores of attention and calculation in MMSE, and lower scores of forward/backward digit span, serial 7 s-administration, verbal fluency, and abstraction in MoCA (all *P* < 0.05, Fig. 1d–i).

**Fig. 1:**
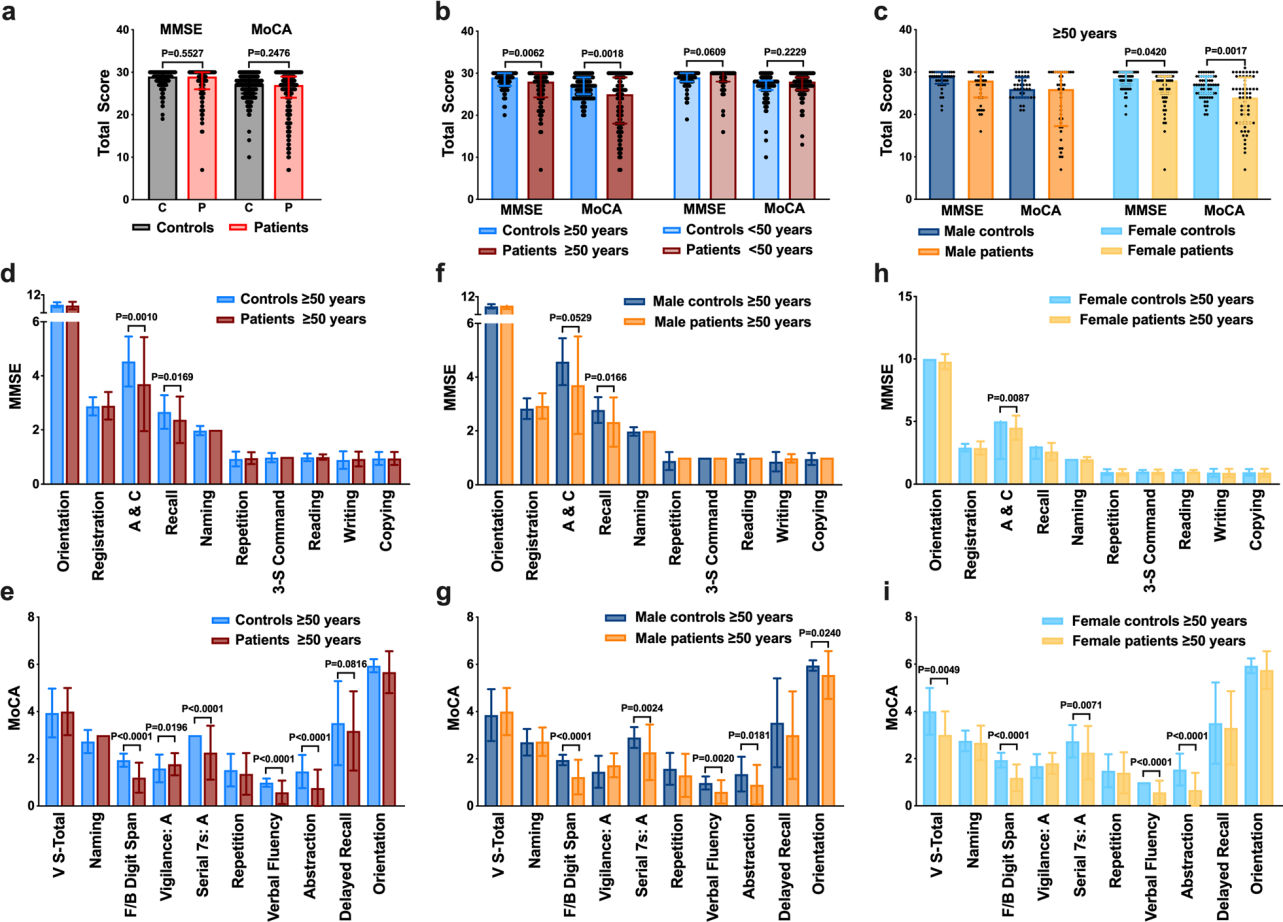
Cognitive dysfunction in patients infected with Omicron variant. **a** MMSE and MoCA total scores in patients infected with Omicron variant and controls.** b** MMSE and MoCA total scores in patients aged ≥ 50 and < 50 years infected with Omicron variant and controls. **c** MMSE and MoCA total scores in males and females aged ≥ 50 with or without Omicron variant infection. **d** Detailed comparison of MMSE items between patients aged ≥ 50 with Omicron variant infection and controls. **e** Detailed comparison of MoCA items between  patients aged ≥ 50 with Omicron variant infection and controls.** f, g** Detailed comparison of MMSE items (**f**) and MoCA items (**g**) between male patients aged ≥ 50 with Omicron variant infection and male controls. **h, i** Detailed comparison of MMSE items (**h**) and MoCA items (**i**) between female patients aged ≥ 50 with Omicron variant infection and female controls. *P < 0.05, **P < 0.01, ***P < 0.001 vs controls. 3-S Command, 3-Stage Command; A & C, Attention and Calculation; F/B Digit Span, Forward/Backward Digit Span; Serial 7 s: A, Serial 7 s: Administration; V S-Total, Visuoconstructional Skills-Total; Vigilance: A, Vigilance: Administration

Although our findings were limited by the small sample size, it is the first study reporting that patients aged over 50 infected with the Omicron BA.2.2.1 variant display cognitive impairment. Therefore, our study suggests the importance and urgency for patients, physicians, and the whole society to pay more attention to the cognitive decline induced by COVID-19 infection in the aging society. Since most patients had no pneumonia or any other severe symptoms, the cognitive impairment may be mediated by peripheral inflammation that induces endothelial disruption, microglial activation, neurotransmitter depletion, and microvascular compromise, and may lead to leukoencephalopathy and focal/global cortical atrophy, resulting in network dysfunction and cognitive changes [5, 6]. Further investigations are needed to clarify the underlying mechanisms.

Notably, MMSE and MoCA were the only screening tools in this study. Being usually the first step to determine cognitive performances, they are not comprehensive, and their insensitivities may be masking effects that require more in-depth studies to reveal. More in-depth studies employing functional MRI and PET-CT will be performed to comprehensively examine the potential alterations of brain function and structure in patients infected with SARS-CoV-2 Omicron variant.

In addition, it is important to examine if the impaired cognitive function of patients infected with SARS-CoV-2 Omicron variant is specific to SARS-CoV-2 or is just a form of expression of sickness behavior [7, 8]. Although patients of the present cohort were asymptomatic or with mild infection symptoms, the majority of them might be having sickness behavior [8]. Here, only patients over 50 years showed cognitive dysfunction, although sickness behavior was observed in patients of both groups in our study. Hence, we may partially exclude the potential influences of sickness behavior/mild discomfort on the cognitive dysfunction of patients in the tests. To confirm the specific effect of SARS-CoV-2 omicron variant infection on cognitive performance and neural circuit functions, long-term influences of infection should be comprehensively monitored in future studies.

## Data Availability

Not applicable.

## Abbreviations

COVID-19: Coronavirus disease 2019
MMSE: Mini-Mental State Examination
MoCA: Montreal Cognitive Assessment
SARS-CoV-2: Severe acute respiratory syndrome coronavirus 2
VOC: Variant of concern
